# In Situ Crosslinked Biodegradable Hydrogels Based on Poly(Ethylene Glycol) and Poly(ε-Lysine) for Medical Application

**DOI:** 10.3390/molecules29225435

**Published:** 2024-11-18

**Authors:** Xia Ding, Bing Yang, Zhaosheng Hou

**Affiliations:** 1 School of Intelligence Engineering, Shandong Management University, Jinan 250357, China; 14438120160212@sdmu.edu.cn; 2 College of Chemistry, Chemical Engineering and Materials Science, Shandong Normal University, Jinan 250014, China

**Keywords:** hydrogels, 4–arm–poly(ethylene glycol), poly(ε–lysine), biodegradable, drug release, antibacterial

## Abstract

Hydrogels have emerged as promising biomaterials due to their excellent performance; however, their biocompatibility, biodegradability, and absorbability still require improvement to support a broader range of medical applications. This paper presents a new biofunctionalized hydrogel based on in situ crosslinking between maleimide-terminated four-arm-poly(ethylene glycol) (4–arm–PEG–Mal) and poly(ε-lysine) (ε–PL). The PEG/ε–PL hydrogels, named LG–n, were rapidly formed via amine/maleimide reaction by mixing 4–arm–PEG–Mal and ε–PL under physiological conditions. The corresponding dry gels (DLG–n) were obtained through a freeze-drying technique. ^1^H NMR, FT–IR, and SEM were utilized to confirm the structures of 4–arm–PEG–Mal and LG–n (or DLG–n), and the effects of solid content on the physicochemical properties of the hydrogels were investigated. Although high solid content could increase the swelling ratio, all LG–n samples exhibited a low equilibrium swelling ratio of less than 30%. LG–7, which contained moderate solid content, exhibited optimal compression properties characterized by a compressive fracture strength of 45.2 kPa and a deformation of 69.5%. Compression cycle tests revealed that LG–n demonstrated good anti-fatigue performance. In vitro degradation studies confirmed the biodegradability of LG–n, with the degradation rate primarily governing the drug (ceftibuten) release efficiency, leading to a sustained release duration of four weeks. Cytotoxicity tests, cell survival morphology observation, live/dead assays, and hemolysis tests indicated that LG–n exhibited excellent cytocompatibility and low hemolysis rates (<5%). Furthermore, the broad-spectrum antibacterial activity of LG–n was verified by an inhibition zone method. In conclusion, the developed LG–n hydrogels hold promising applications in the medical field, particularly as drug sustained-release carriers and wound dressings.

## 1. Introduction

Hydrogels are three-dimensional networks of hydrophilic polymers that have high water-retaining capabilities [1,2]. They can maintain structural integrity while swelling in aqueous medium or physiological fluids. The flexibility, tunable mechanical properties, and favorable biocompatibility make them ideal for various medical applications, including drug delivery [3,4], cell culture [5], tissue engineering [6,7,8], and wound healing [9,10]. In recent years, smart hydrogels with external triggers (temperature, electricity, magnetism, pH, light, etc.) have been developed for targeted-release drug delivery systems, which can effectively reduce the side effects of drugs [11,12,13]. Many strategies are explored to prepare hydrogels, including chemical reactions, and light- and heat-induced polymerization reactions [14,15]. However, these approaches have drawbacks, including potential toxicity, delayed gelation, or requiring supplementary devices [16]. Moreover, most of the produced hydrogels exhibit instability and a concurrent loss of bioactivity due to their uncontrolled gelation chemistries [17,18]. Therefore, developing a simple and rapid method to prepare biodegradable hydrogels with high biocompatibility and biostability is significant for broadening their applications in the medical field.

Poly(ethylene glycol) (PEG) is a versatile nonionic polymer known for its high biocompatibility, superior hydrophilicity, and non-toxicity [19]. The adjustment of its molecular weight and structure enables tailored applications in various pharmaceutical and medical areas, such as in drug delivery systems to enhance solubility and bioavailability, in tissue engineering as scaffolds for cell growth, and in oncology through PEGylation to reduce immunogenicity [20,21,22]. PEG-based hydrogels exhibited exceptional efficacy in guiding the behavior of encapsulated cells due to their inherent resistance to protein adsorption [23,24]. Recently, hydrogel systems made from multi-arm PEGs or their derivatives have been designed as promising scaffolds for applications in tissue engineering [25,26].

ε-Poly(L–lysine) (ε–PL), a natural cationic antibacterial peptide, is composed of ε–lysine by isopeptide bonds [27,28]. This compound exhibited various beneficial properties, including biocompatibility, biodegradability, water solubility, thermal stability, edibility, and antibacterial effects [29,30]. The body can rapidly metabolize ε–PL through its enzyme system into the monomer ε–lysine, which is subsequently absorbed, thereby minimizing the potential for accumulation and toxicity. Furthermore, the antibacterial characteristics of ε–PL effectively inhibit the growth and reproduction of various microorganisms, which reduces the risk of infection and improves the safety of medical devices [31,32]. Consequently, ε–PL presents a promising biomedical material for various applications, such as antibacterial surface coatings, drug delivery systems, and protective films [33,34,35]. However, the polycationic nature of ε–PL can lead to non-specific interactions with cell membranes, potentially impacting drug targeting [36]. Additionally, its relatively rapid degradation rate could influence the effectiveness of long-term drug release.

In this paper, in situ crosslinked hydrogels were developed through the amine/maleimide reaction between maleimide-terminated four-arm-poly(ethylene glycol) (4–arm–PEG–Mal) and ε–PL under physiological conditions for medical application. 4–Arm–PEG–Mal was first synthesized from a commercially available 4–arm−PEG−OH. Subsequently, PBS solutions of 4–arm–PEG–Mal and ε–PL were mixed and sprayed using a double-barrel syringe to rapidly produce hydrogels with varying solid content. The corresponding dry gels were obtained through freeze drying. The effect of the solid content in hydrogels on their physicochemical characteristics (thermal properties, mechanical properties, swelling behavior, degradation performance, and drug release properties) was investigated. The biocompatibility of the hydrogels was preliminarily evaluated through cytotoxicity tests, cell survival morphology observation, live/dead assays, and hemolysis tests. Additionally, the antibacterial properties of the hydrogels were assessed using the inhibition zone method.

## 2. Results and Discussion

### 2.1. Preparation of LG-n Hydrogels

**Figure 1:**
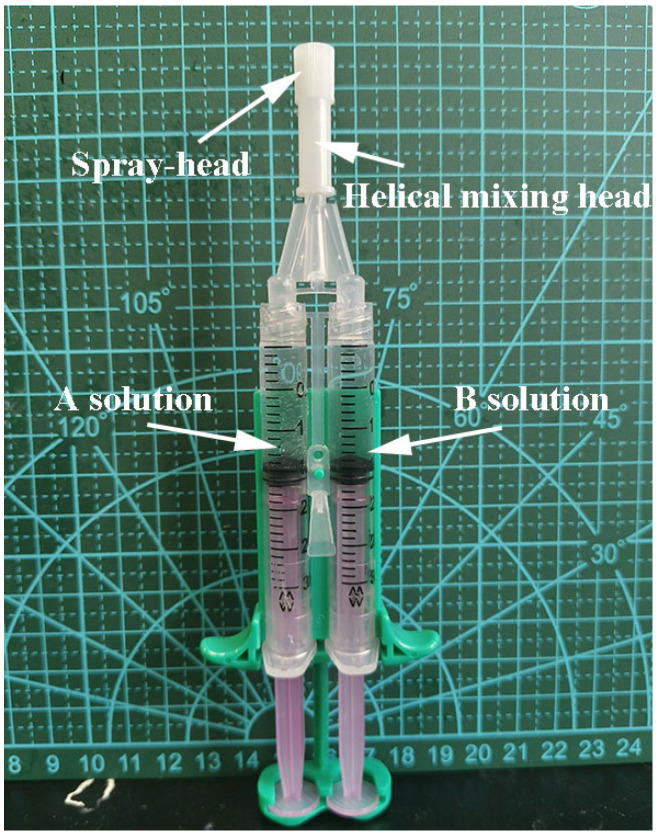
Photo of the dual–chamber syringe equipped with a helical mixing head.

Due to the electrophilicity of 4–arm–PEG–Mal and nucleophilicity of ε–PL, the electrophilic-nucleophilic substitution reactions easily occurred under mild conditions by mixing their solution at different pH values, resulting in the formation of crosslinked PEG/ε–PL hydrogels (LG-n). Direct mixing of the two solutions yielded an inconsistent mixture, which compromised the hydrogel formation process. To address this, a double syringe equipped with a helical mixing head (Figure 1) was adopted to facilitate complete reactions between the two components, thereby promoting the formation of a homogeneous hydrogel. It provided a more controlled, efficient, and scalable method for hydrogel formation compared to direct mixing methods. After the 15 s of spraying, the mixture exhibited negligible flow, and by 40 s, the hydrogel had formed essentially.

### 2.2. FT-IR Spectra

**Figure 2:**
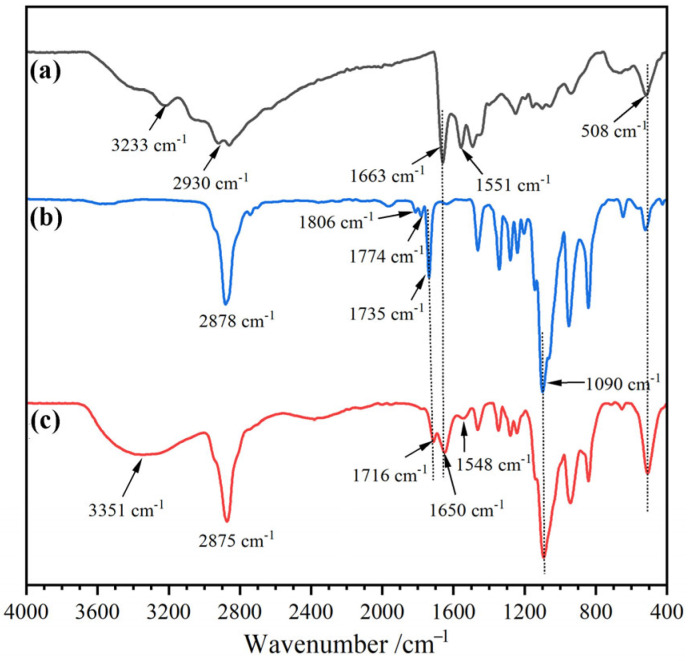
FT–IR spectra for (**a**) ε–PL, (**b**) 4–arm–PEG–Mal, and (**c**) DLG–7.

The chemical structures of ε–PL, 4–arm–PEG–Mal, and dry PEG/ε–PL gels (DLG–n) were characterized by FT-IR, and the spectra are displayed in Figure 2 (DLG–n samples have similar FT-IR spectra, and DLG–7 is taken as a representative sample). In the FT-IR spectrum of ε–PL (Figure 2a), the absorption peaks at 3233, 2930, 1663, 1551, 1250, and 508 cm^−1^ belonged to the stretching vibrations of N–H, C–H, amide I, amide II, C–N, and –NH–, respectively. In addition, a broad strong absorption was observed at 3000~2800 cm^−1^, which was attributed to the asymmetric and symmetric stretching vibrations of –NH_3_^+^ [37]. 4–arm–PEG–Mal (Figure 2b) exhibited the characteristic absorption of –CH_2_–, ester C=O, and ether C–O–C at 2870, 1735, and 1090 cm^−1^, respectively. The weak peaks at 1774 and 1806 cm^−1^ were ascribed to the characteristic absorption of the C=O and amide groups in the maleimide ring. The maleimide groups were linked only at the end of the PEG chain, and their low content resulted in reduced absorption intensity, proving the successful synthesis of 4–arm–PEG–Mal. In the FT–IR spectrum of DLG–7 (Figure 2c), the characteristic peaks of ε–PL and 4–arm–PEG–Mal were observed. The absorption peaks of –CH_2_–, ester C=O, amide I, amide II, ether C–O–C, and –NH– appeared at 2875, 1716, 1650, 1548, 1090, and 508 cm^−1^, respectively. The absorption intensity of –NH_3_^+^ (3000~2900 cm^−1^) showed a significant decrease, accompanied by a broader band of amide (508 cm^−1^), which confirmed that the chemical reactions of maleimide/–NH_2_ indeed occurred and the cross-linked structures were formed. It was worth noting that the absorption peaks for ester C=O and amide I exhibited a blue shift, due to the H–bonds formed between C=O and –NH–. A similar phenomenon was also observed in our reported poly(ether-urethane) elastomers based on phenol-urethane bonds [38]. Additionally, the broad peak at 3351 cm^−1^ corresponded to the absorption of residual moisture in the dry gel.

### 2.3. Microstructure

**Figure 3:**
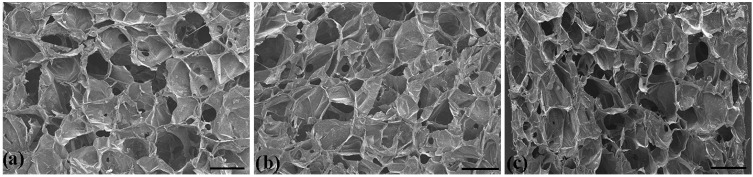
SEM photos of (**a**) DLG–3, (**b**) DLG–7, (**c**) DLG–11 (scale bar: 500 μm).

The SEM images of DLG–n with various solid content are presented in Figure 3. A porous structure with significant interconnectivity was observed across all samples [39]. Twenty pores were selected from each SEM image to measure the pore sizes (Photoshop CS6™) and pore size distribution (Origin 2020™). The average pore sizes were 498 ± 71, 454 ± 65, and 394 ± 59 μm for DLG–3, –7, and –11, respectively. The pore size gradually decreased with the increase in solid content in the hydrogels, which correspondingly indicated a reduction in the density of the samples. The dense pores enhanced the contact surface between the dry gel and water molecules, thereby facilitating water retention in the hydrogel. Furthermore, this structure effectively dispersed the external forces, thus preserving the shape of the hydrogels and enhancing their anti-compressive properties [40]. The porous network structure contributed to excellent water absorbency and favorable mechanical properties.

### 2.4. TGA Analysis

**Figure 4:**
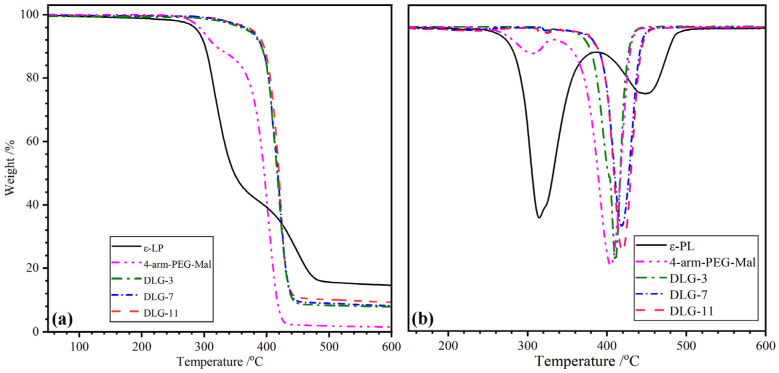
(**a**) TGA and (**b**) DTG curves of ε–PL, 4–arm–PEG–Mal, and DLG–n.

Figure 4 shows the TGA and DTG curves of ε–PL, 4–arm–PEG–Mal, and DLG–n, and the characteristic values derived from the curves are listed in Table 1. The pyrolysis process of ε–PL could be divided into two weight-loss stages, which was consistent with the reported result [41]. The first stage occurred at low temperatures with a maximum temperature (T_1_) of 315.0 °C belonged to the thermal decomposition of the amide bond in the main chain. The slight weight loss in the second stage with T_2_ of 449.6 °C originated from the decomposition of the organic salt in the side chain. The heat-resistant organic salts resulted in a residual weight (W_r_) of up to 14.6% at the end of the test. A two-step weight loss was found in the thermal decomposition curves of 4–arm–PEG–Mal. The slight mass loss at 306.0 °C (T_1_) was attributed to the pyrolysis of ester bonds, and the main mass loss at 405.6 °C (T_2_) belonged to the decomposition of ether bonds in the backbone [42]. DLG–n with different solid content exhibited overlapped TGA curves, meaning similar thermal stability. It could be concluded that the solid content had little effect on the thermal stability of the dry gel. Only one weight-loss peak with T_2_ ranging from 411.2 to 421.0 °C was found in the DTG curves of DLG–n, manifesting good component compatibility. The T_5%_ of DLG–n appeared at ~370 °C, which was much higher than that of ε–PL (289.3 °C) and 4–arm–PEG–Mal (295.4 °C). The high initial thermal stability of DLG–n should be ascribed to the three-dimensional network structure linked by covalent amide bonds and noncovalent H–bonds, which needed more energy to decompose. It indicated that crosslinking improved the heat resistance of DLG–n and a T_5%_ higher than 200 °C meant that the dry gels were suitable for high-temperature sterilization.

### 2.5. DSC Analysis

**Figure 5:**
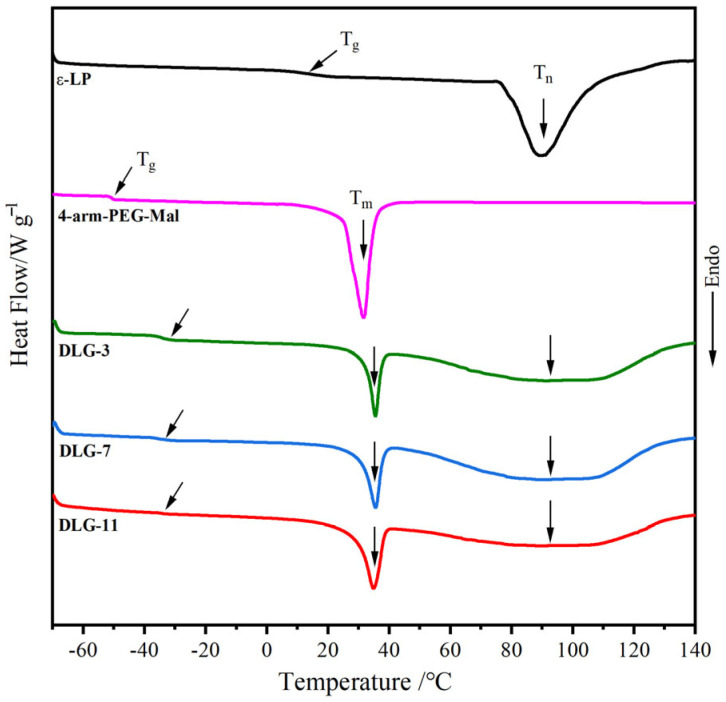
DSC curves of ε–PL, 4–arm–PEG–Mal, and DLG–n.

The DSC curves for ε–PL, 4–arm–PEG–Mal, and DLG–n are presented in Figure 5, and the thermal transition values are summarized in Table 2. ε–PL exhibited a slightly higher glass transition temperature (T_g_) at 13.4 °C, which was due to the NH_3_^+^ in the side chain. As reported in the published paper, the T_g_ of 4–arm–PEG–Mal appeared at a low temperature of −50 °C, owing to its highly flexible ether bonds [43]. Only one T_g_ was observed in the crosslinked DLG–n curves, indicating good compatibility between PEG and ε–PL. The crosslinked network structure reduced the flexibility of the ether bonds, resulting in higher T_g_ values than 4–arm–PEG–Mal. The T_g_ values of approximately ~−34 °C meant that DLG–n chains were in a highly flexible state at normal temperature, which was a crucial factor for enabling the mechanical properties of the hydrogels. In addition, both ε–PL and 4–arm–PEG–Mal displayed a distinct crystal melting endothermic peak, appearing at 76–110 °C (T_n_ = 89.3 °C, Δ*H*_n_= 43.9 J·g^−1^) and 24–37 °C (T_m_ = 31.7 °C, Δ*H*_m_= 28.1 J·g^−1^), respectively. Both endothermic peaks appeared in the DSC curves of DLG–n; however, the corresponding Δ*H* values were significantly lower than those of the raw materials. This reduction could be attributed to the crosslinking, which hindered the orderly arrangement of chains and decreased crystallization capacity. The similar DSC curves of DLG–n demonstrated that the solid content did not affect their thermal transition properties.

### 2.6. Swelling Performance

**Figure 6:**
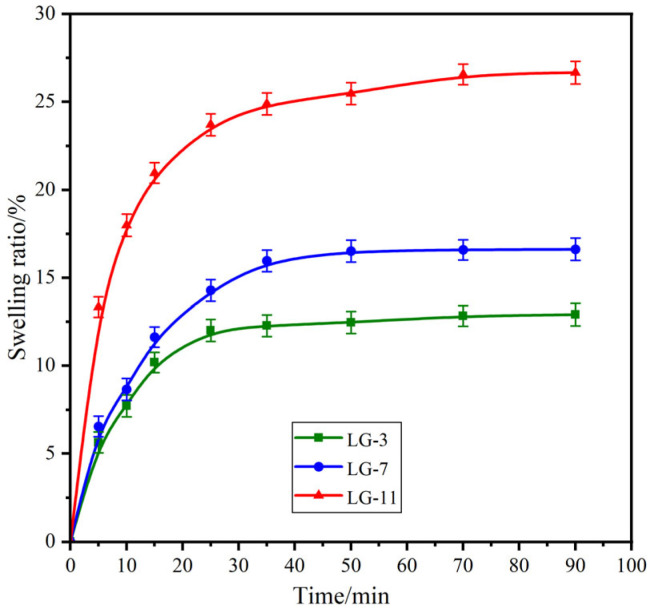
Swelling curves of ε–PL, 4–arm–PEG–Mal, and DLG–n (*n* = 3).

Swelling studies were conducted to investigate the influence of solid content on the performance of the prepared hydrogels. The swelling behaviors of the LG–n with different solid contents were explored over time, and the resulting swelling curves are presented in Figure 6. All samples exhibited a rapid initial increase in SR, followed by gradually slowing down and achieving equilibrium. The time required for LG–3, –7, and –11 samples to reach swelling equilibrium was 25, 50, and 70 min, respectively. The corresponding equilibrium swelling rates (ESRs) for these samples were 11.8%, 16.5%, and 26.4%. The results indicated that with an increase in solid content in hydrogels, both the SR and ESR exhibited a significant rise. This phenomenon can be attributed to two primary factors [44]: one is the hydrophilicity of the PEG segment, which increases with the rising solid content; another is the tighter network structure formed by the higher solid content. Consequently, high solid content could enhance the water–holding capacity of the hydrogel, resulting in a slower SR and a higher ESR. It should be noted that LG–n hydrogels with different solid content presented low swelling properties with ESR values less than 30%.

### 2.7. Compression Performance

**Figure 7:**
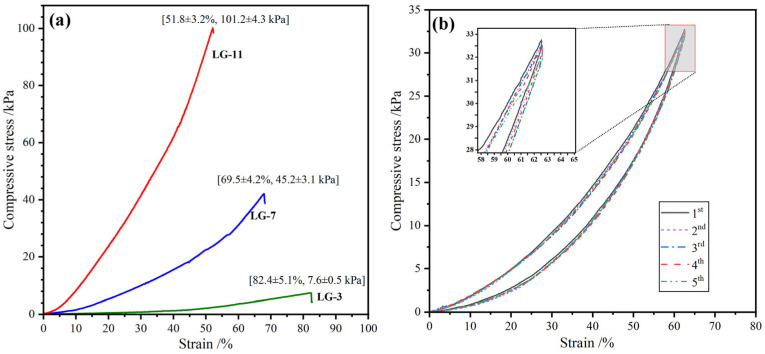
(**a**) Compressive stress–strain profiles of LG–n hydrogels and (**b**) cyclic compressive stress–strain profiles of LG–7 hydrogels.

The compressive performance of hydrogels is of paramount importance, as it dictates their capacity to withstand external stress and maintain structural integrity in practical applications. The compressive stress–strain profiles of the covalently crosslinked LG–n are expressed in Figure 7a. As expected, the compressive strength (σ) increased with the increase in compressive deformation (ε). When the solid content increased from 3% to 11%, the fracture strength (σ_f_) of LG–n hydrogels increased rapidly from 7.6 to 101.2 kPa, while the fracture deformation (ε_f_) decreased gradually from 82.4 to 51.8%. The higher solid content could produce more crosslinking points in the hydrogel and form a denser network structure, which needed more energy against external pressure [45]. However, a higher solid content could enhance the σ_f_; excessively high solid content led to the hydrogel becoming more brittle. Therefore, to obtain the optimum comprehensive performance, the solid content of LG–n hydrogels should be maintained at a level not exceeding 11 wt%.

To further illustrate the mechanical properties of the hydrogels, the cyclic compressive test was performed to investigate their anti-fatigue performance. Taking LG–7 as a representative sample, the ε was set as 90% of the ε_f_. Five successive loading–unloading tests were carried out, and the stress–strain profiles are displayed in Figure 7b. It was found that although the maximum compressive strength (σ_m_) exhibited a slight decrease with the increase in the cycle number, the stress recovery rates (the percentage of σ_m_ to the original value [46]) were higher than 95%. In addition, the compressive hysteresis loops almost overlapped during the five cycles, demonstrating that the hydrogels could rapidly recover their pristine shape after removing the external force [47]. The results indicated that the hydrogels possessed excellent elastic resilience and fatigue resistance.

### 2.8. Biodegradability

**Figure 8:**
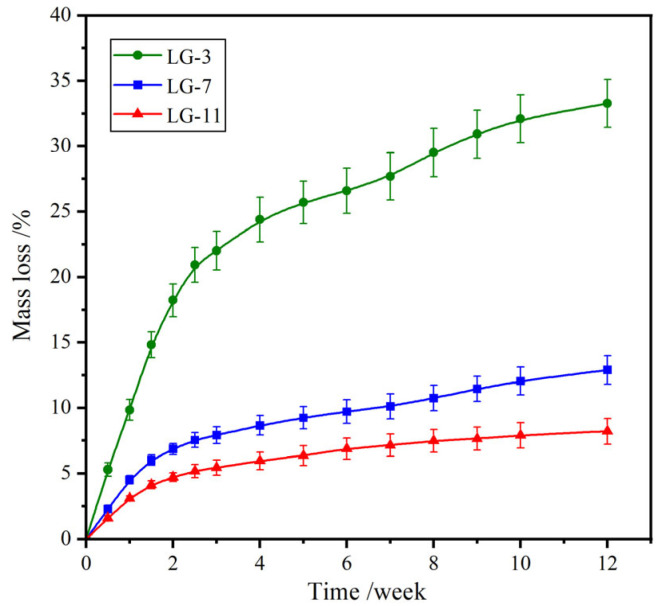
Mass loss curves of LG–n against degradation time in PBS (pH 7.4) at 37 °C (*n* = 3).

After serving a temporary application in the body, the medical hydrogels are expected to degrade, with degradation products being absorbable or metabolizable by the living body. In vitro degradation of the fabricated LG–n hydrogels was carried out in PBS (pH 7.4) at 37 °C using a gravimetric method, and the time-dependent mass loss is shown in Figure 8. All samples showed a rapid mass loss during the first two weeks, which was due to the dissolution of the uncrosslinked molecular chains. After degradation for 12 weeks, the mass loss for LG–3, –7, and –11 was 33.3%, 12.9%, and 8.2%, respectively. The degradation rate reduced with the increase in solid content. Higher solid content contributed to the formation of more compact crosslinked networks, which restricted the contact of water molecules with hydrolyzable amide and ester bonds, resulting in enhanced hydrolytic stability. Thus, the degradation rate of LG–n could be controlled by adjusting the solid content. It was inferred that the final hydrolytic degradation products of the hydrogels were PEG and ε–lysine, which could be absorbed and metabolized by the body, respectively.

### 2.9. Drug Release Performance

**Figure 9:**
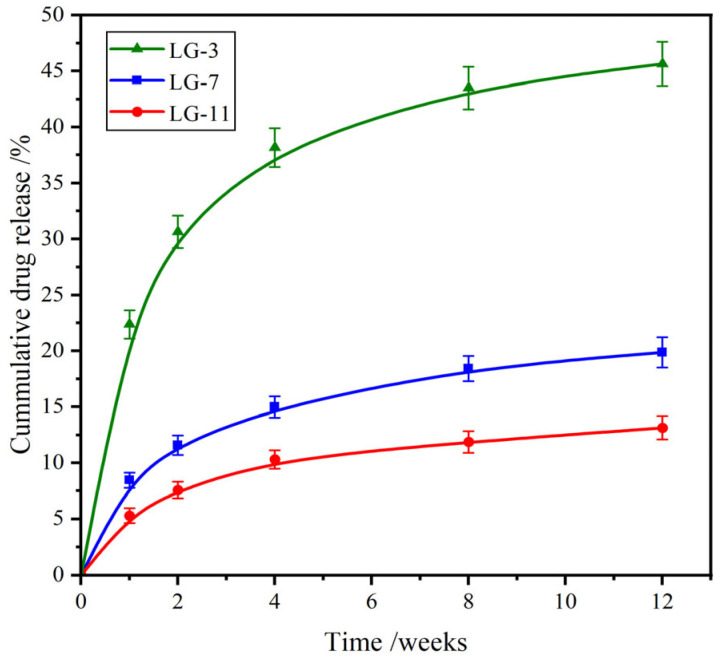
Drug release curves from LG–n hydrogels in PBS (pH 7.4) at 37 °C (*n* = 3).

Ceftibuten (CFB), a third-generation cephalosporin antibiotic, is effective against a wide range of GP and GN bacteria. It is recognized for its favorable pharmacokinetic properties and remains widely used in the treatment of respiratory tract infections, otitis media, and uncomplicated urinary tract infections [48,49]. Figure 9 shows the in vitro drug release profiles of CFB from LG–n in PBS (pH 7.4) at 37 °C. Relatively high drug release rates were observed in the initial two weeks, which was the burst release of drug molecules attached to the surface of the hydrogel. After that, the rate gradually slowed down with time. Because the amide groups of CFB could form H–bonds with the amide groups of the crosslinked PEG/ε–PL chain, it was difficult for drug molecules to diffuse out of the hydrogel. Hence, the drug release rate in this stage was primarily controlled by the degradation of the hydrogel. After incubation for 12 weeks, the cumulative drug release (CDR) of LG–3, –7, and –11 was 45.8%, 19.8%, and 12.9%, respectively. Therefore, the effective drug release of LG–n was maintained for more than four months, and the fabricated hydrogels showed a prospective application for long-term drug delivery systems.

### 2.10. Cytocompatibility Assay

**Figure 10:**
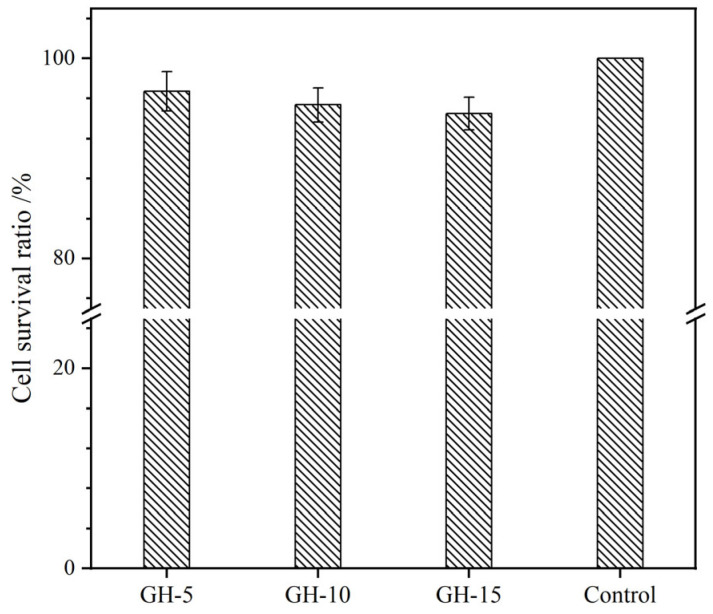
Cell survival rate in LG–n hydrogel extracts by MTT (37 °C, 72 h, *n* = 3).

**Figure 11:**
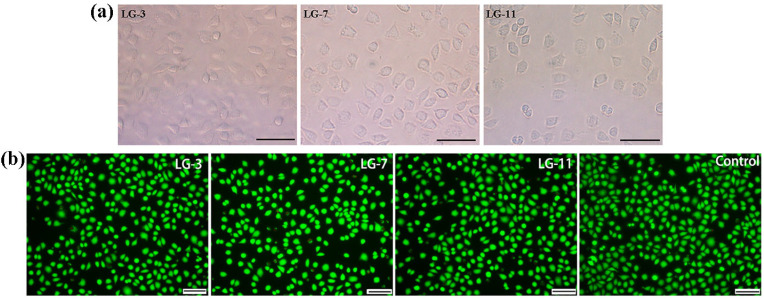
(**a**) Cell morphologies on LG–n hydrogel surface (37 °C, 72 h, scale bar: 100 μm) and (**b**) live/dead cells in LG–n extracts (37 °C, 72 h, scale bar: 100 μm).

Cytocompatibility is known as the principal step in estimating the biocompatibility of biomaterials. Here, the cell relative growth rate (RGR), survival state, and live/dead activity were adopted to assess the cytocompatibility of LG–n hydrogels with fibroblast L929 as the test cells. Figure 10 presents the RGR of cells cultured in hydrogel extracts for 72 h, as determined by the MTT assay. All the samples exhibited RGR values exceeding 95%, indicating that the solid content had minimal impact on the RGR. Based on ISO 10993–6:2007 standards [50], the cytotoxicity was class 1 and could meet the requirements for in vivo biomaterials. The optical images of cells cultured on the surface of LG–n for 72 h are displayed in Figure 11a. A large number of cells adhered to the surface of the hydrogel and presented an entirely spread morphology with elongated shapes, which manifested that the cells exhibited good survival states [51]. A cell live/dead activity test was performed on the cells cultured with LG–n extracts, and the micrographs are shown in Figure 11b. It was observed that the vast majority of cells remained viable with few dead cells. The survival status of the cells was kept consistent with the control sample. All the results confirmed that LG–n hydrogels possessed excellent cytocompatibility.

### 2.11. Hemolysis Assay

**Figure 12:**
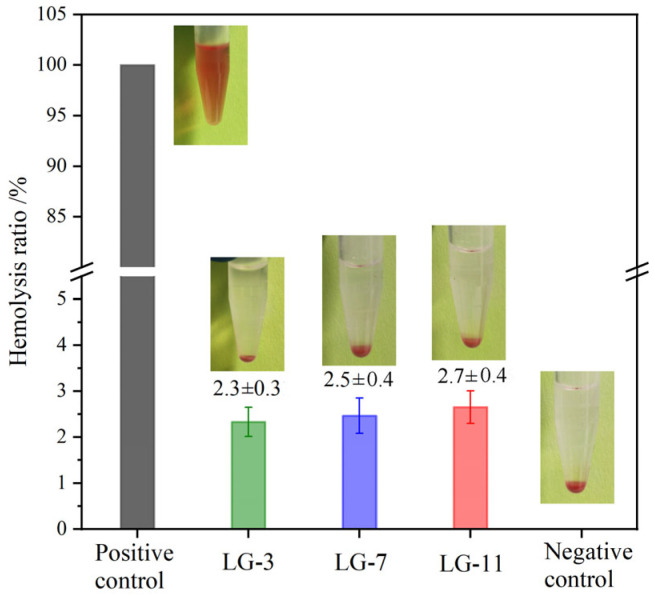
Hemolysis test images and hemolysis rates of hydrogel LG–n (37 °C, 24 h, *n* = 3).

It is difficult for medical materials to avoid contact with blood, and in vitro hemolysis testing is a simple method for evaluating their hemocompatibility in direct contact with blood [52]. The hemolysis tests were performed with anticoagulant rabbit blood, and the hemolysis rates (HRs) and hemolysis effect images for LG–n hydrogels are shown in Figure 12. The supernatant from the positive control displayed a distinct red color, which was attributed to the release of protoheme from the ruptured red blood cells. In contrast, all hydrogel groups appeared transparent with a yellowish hue, which was similar to the negative control. With the HR of the positive control set at 100%, the HR values for LG–3, –7, and –11 were 2.3%, 2.5%, and 2.7%, respectively. Although the HR values exhibited a slight rise with the increase in solid content, they were all lower than 5%, demonstrating that the prepared hydrogels had a low hemolytic effect and good hemocompatibility.

### 2.12. Antibacterial Property

**Figure 13:**
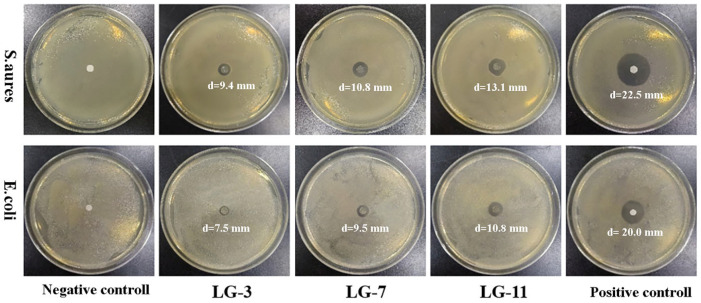
Antibacterial activities of LG–n against *E. coli* and *S. aureus*.

During the wound-healing process, bacterial infections can lead to persistent inflammation or other complications, thus, the antibacterial capacity in biomedical hydrogels is conducive to the healing of wounds. The antibacterial activity of LG–n hydrogels against *E. coli* and *S. aureus* was measured by the inhibition zone method, and the results are presented in Figure 13. The absence of an inhibition zone in the negative control indicated no antibacterial activity. Obvious inhibition zones were formed around the LG–n, demonstrating their effectiveness in resisting the growth of *E. coli* and *S. aureus*. The antibacterial capacity increased gradually with the increase in solid content in hydrogels, which was reflected by the increased diameter of the inhibition zone from LG–3 to LG–11. The antibacterial activity was primarily attributed to the residual –NH_2_ of ε–PL, which could interact with peptidoglycan on the bacterial cell wall [53]. Tan’s groups demonstrated that ε–PL could induce the structural change in peptidoglycan, which led to the destruction of the cell wall, the release of intracellular fluid, and the death of bacteria [54]. The larger inhibition zones were achieved against *E. coli* than *S. aureus*, indicating higher antibacterial activity of LG–n for GN bacteria than GP bacteria. This should be due to the thicker peptidoglycan layers and external lipid membranes in GP bacteria [55]. These results suggested that the broad-spectrum antibacterial LG–n hydrogels could reduce the chance of bacterial infection and showed great potential for promoting wound healing.

## 3. Materials and Methods

### 3.1. Materials

4–Arm–PEG–OH (*M_n_* = 10 kDa) was obtained from Sigma-Aldrich (Shanghai, China) and vacuum dehydrated at 85 °C for 3.5 h before use. Glutaric anhydride (GA, 99%), 4–dimethylaminopyridine (DMAP, 95%), N–hydroxymaleimide (NHM, 97%), triethylamine (TEA, 99%), and 1–(3–dimethylaminopropyl)–3–ethylcarbodi imide (EDC, 97%) were purchased from Aladdin (Shanghai, China). ε–PL hydrochloride (*M_n_* = 5.0 kDa) was acquired from Macklin (Shanghai, China). CFB (99.5%) was supplied by Sihuan Pharmaceutical Co., Ltd. (Jinan, China). L929 Mouse fibroblasts (1 × 10^6^ cells/T25) and fresh rabbit blood were acquired from Puyi Biotechnology Co., Ltd. (Shanghai, China). Other reagents were AR grade.

### 3.2. Synthesis of 4–Arm–PEG–Mal

**Figure 14:**
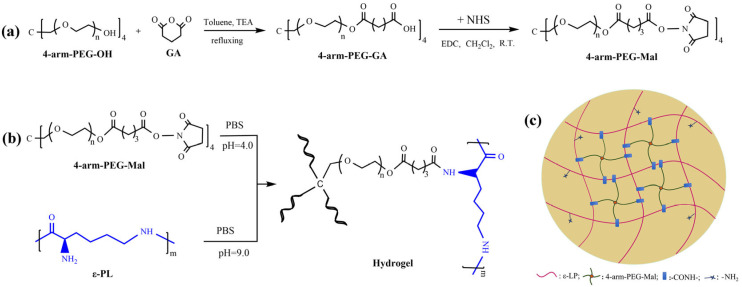
(**a**) Synthesis of 4–arm–PEG–Mal, (**b**) preparation of hydrogel LG–n, and (**c**) crosslinking schematic diagram of hydrogel LG–n.

4–Arm–PEG–Mal was synthesized according to the reported method with slight modifications [56], as shown in Figure 14a. Briefly, 4–arm–PEG–OH (1.5 mmol), GA (18.0 mmol), and TEA (18.0 mmol) were dissolved in 150 mL of toluene. After refluxing for 24 h, the solution was concentrated to ~30 mL, which was then precipitated by diethyl ether (200 mL, −5 °C). The obtained crude product was dissolved in CH_2_Cl_2_ and precipitated again with diethyl ether. After filtration and vacuum drying at room temperature, the carboxyl–terminated 4–arm–PEG (4–arm–PEG–GA) was obtained with a yield of 82.4%. 4–arm–PEG–GA (1.0 mmol) and NHM (18.5 mmol) were dissolved in 100 mL of CH_2_Cl_2_, and a CH_2_Cl_2_ solution (30 mL) of EDC (18.5 mmol) was added dropwise to the system under stirring. After stirring for 24 h, the mixture was filtered, and the filtrate was concentrated to ~30 mL. The diethyl ether (200 mL, −5 °C) was added to precipitate the solution. The same purification procedure was performed twice, followed by vacuum drying at room temperature to produce 4–arm–PEG–Mal with a yield of 84.4%.

The chemical structure of 4–arm–PEG–Mal was confirmed by ^1^H NMR. ^1^H NMR (600 MHz, CDCl_3_, ppm): δ 4.21 (t, –CH_2_*–CH*_2_–COO–), 3.80–3.44 (m, –*CH*_2_*–CH*_2_–O–), 3.38 (s, C*–CH*_2_–O), 2.84 (br, N–CO–*CH*_2_–), 2.72 (t, OOC*–CH*_2_–CH_2_–), 2.50 (t, –CH_2_–*CH*_2_–COO–N), 2.07 (m, –CH_2_–*CH*_2_–CH_2_–). Substitution degree of end groups: S_δ2.07_:S_δ3.38_ = 0.93:1.01 = 92.1%.

### 3.3. Preparation of PEG/ε–PL Hydrogels

The PEG/ε–PL hydrogels (LG–n) were crosslinked by amine/maleimide substitution reaction; the preparation process and crosslinking schematic diagram of PEG/ε–PL hydrogels are shown in Figure 14b and Figure 14c, respectively. The molar ratio of maleimide groups to amino groups was fixed at 1:1.5. 4–Arm–PEG–Mal and ε–PL were dissolved in PBS with pH values of 4 (A solution) and 9 (B solution), respectively. The solutions were placed separately in the injection tube of a dual-chamber syringe, quickly mixed by the helical mixing head, and sprayed. The mixture was cured for ~3 min at ambient temperature to form PEG/ε–PL hydrogel, denoted as LG–n (n is the solid content of 3%, 7%, and 11%). The corresponding dry gels named DLG–n were obtained by the freeze-drying method.

### 3.4. Instruments and Characterization

The ^1^H NMR spectrum was measured using an AVANCEII600MHz spectrometer (Bruker, Billerica, MA, USA) with CDCl_3_ as the solvent. The FT–IR spectrum was recorded on an ALPHAII infrared spectrometer (Bruker). The dry gel was brittle-fractured in liquid nitrogen, and the cross-sectional microstructure was observed by SEM (SU–8010, Hitachi, Tokyo, Japan). The thermostability of the dry gel was determined using a TGA 2050 (Universal, New Brunswick, NJ, USA) under N_2_ atmosphere with a heating rate of 20 °C/min. DSC 2910 (TA, New Castle, DE, USA) was adopted to measure the thermal transition, and the heating rate was set at 10 °C/min.

The swelling behavior of the hydrogel was assessed gravimetrically by measuring the mass of the absorbed water over time. The cylindrical samples (diameter: 15 mm; height: 5 mm) were weighed and immersed in deionized water at 37 °C. At a fixed time, the swollen sample was fetched out. The water on the surface was removed with filter paper and then weighed. The swelling ratio (SR) was calculated according to the formula: SR (%) = (M_s_ − M_o_)/M_o_ × 100, where M_s_ and M_o_ are the mass of swollen and pristine hydrogels.

The compressive stress–strain curves were obtained on cylindrical hydrogel samples (diameter: 15 mm; height: 10 mm) using a CT3 texture analyzer (Brookfield, Middleboro, MA, USA) with a TA10A probe. The compression rate was set at 0.05 mm/s and the test was conducted at room temperature.

In vitro degradability of the hydrogels was evaluated by measuring their weight-loss rate in PBS solution (pH 7.4). A wet mesh bag containing the swollen hydrogel sample was weighed (W_o_) and immersed in PBS. After incubation at 37 ± 0.1 °C for a determined time, the mesh bag was removed from PBS, left hanging to drain off the surface water, and weighed (W_d_). The degradation rate (DR) was calculated using the formula: DR(%) = (W_o_ − W_d_)/W_o_ × 100.

The drug sustained-release behavior of hydrogel was carried out according to the reported method with CFB as the model drug [57]. The drug-loaded hydrogels were prepared using the same process, except for adopting PBS solutions containing CFB (1.0 mg/mL).

The cytotoxicity assays, cell survival morphologies, and live/dead measurements were used to evaluate the cytocompatibility of the hydrogels. L929 cells served as the test cells. The cytotoxicity was performed according to the MTT method described elsewhere [58,59]. The morphology of the cells on the hydrogel surface after incubation for 72 h was observed on an inverted microscope. Calcein AM/PI staining kit (Merck, Darmstadt, Hessen, Germany) was adopted to perform the cell live/dead measurement. The cell was first cultured in hydrogel extracts for 72 h. The culture medium was subsequently sucked out, and the Calcein AM/PI staining solution was introduced. After incubation in the dark for 30 min at 5% CO_2_ and 37 °C, the live (green, E_x_/E_m_ = 494/517 nm) and dead (red, E_x_/E_m_ = 535/617 nm) cells were observed under an inverted fluorescence microscope.

The hemolytic behavior of hydrogel was determined according to the requirements of GB/T 14233-2:2005 [60]. The experimental blood was collected from the hearts of healthy rabbits, and sodium citrate (5%) was added to avoid coagulation. The anticoagulated blood (8.0 mL) was diluted with normal saline (NS, 10.0 mL). The tube containing hydrogel (2.0 g) and NS (10 mL) was incubated at 37 °C for 30 min, followed by adding 0.2 mL of diluted blood and incubating for 60 min. After centrifuging for 5 min, the absorbance of the supernatant was obtained by an enzyme-linked immunosorbent assay at 540 nm. The NS and distilled water without hydrogel served as negative and positive controls, respectively. The HR was calculated according to the formula HR (%) = [(A_s_ − A_n_)/(A_w_ − A_n_) × 100, where A_s_, A_n_, and A_w_ were the absorbance values of the sample, negative control, and positive control, respectively.

The inhibition zone method was adopted to assess the antibacterial activity of hydrogel with Gram-negative (GN) *E. coli* and Gram-positive (GP) *S. aureus* as test bacteria. Filter papers soaked in ε–PL (5.0 mg/mL) solution and PBS solution (pH 7.4) were used as a positive and negative control, respectively. After a 24-h incubation at 37 °C, a digital camera was used to record the antibacterial effect, and the diameter of the inhibition zone was measured.

## 4. Conclusions

This paper presents an in situ crosslinked hydrogel (LG–n) developed through the amine/maleimide reaction between 4–arm–PEG–Mal and ε–PL under physiological conditions. The effects of solid content on the physicochemical properties of the hydrogels were investigated. Although a high-solid content could increase the swelling ratio, all LG–n samples demonstrated an equilibrium swelling ratio of less than 30%. LG–7 with moderate solid content exhibited optimal compression properties with a σ_f_ of 45.2 kPa and ε_f_ of 69.5%. Compression cycle tests revealed that LG–n exhibited good antifatigue performance. In vitro degradation studies confirmed the biodegradability of LG–n, with the degradation rate primarily governing the drug (CFB) release efficiency, resulting in a sustained-release duration of four weeks. Cytotoxicity tests, cell survival morphology observation, live/dead assays, and hemolysis tests indicated that LG–n displayed excellent cytocompatibility and low hemolytic effects (HR < 5%). Furthermore, the broad-spectrum antibacterial activity of LG–n was validated by the inhibition zone method. The biodegradable LG–n hydrogels demonstrated superior compression and antifatigue properties, excellent cytocompatibility, and favorable hemocompatibility, suggesting their promising applications in the medical field, particularly as drug sustained-release carriers and wound dressings.

## Figures and Tables

**Table 1:** Characteristic values of ε–PL, 4–arm–PEG–Mal, and DLG–n from TGA and DTG curves.

Samples	T_5%_/°C	T_1_/°C	T_2_/°C	W_r_/%
ε–PL	289.3	315.0	449.6	14.6
4–arm–PEG–Mal	295.4	306.1	405.6	1.6
DLG–3	377.8	/	411.2	8.2
DLG–7	371.8	/	418.6	7.9
DLG–11	370.3	/	421.1	7.8

**Table 2:** Characteristic values of ε–PL, 4–arm–PEG–Mal, and DLG–n from DSC curves.

Samples	T_g_/°C	T_m_/°C	T_n_	Δ*H*_m_/J·g^−1^	Δ*H*_n_/J·g^−1^
ε–PL	13.4	–	89.3	–	43.9
4–arm–PEG–Mal	−49.6	31.7	–	28. 1	–
LG–3	−33.9	45.6	97.1	15.2	25.2
LG–7	−33.9	45.6	97.4	14.7	26.2
LG–11	−33.8	45.5	96.9	14.6	25.9

## Data Availability

All data are contained within the manuscript and are available upon request.
